# Self-Regulation in Eating Behaviors: The Role of Executive Function in Response to Food Stimuli

**DOI:** 10.3390/nu16142318

**Published:** 2024-07-18

**Authors:** Francesca Favieri, Renata Tambelli, Eunice Chen, Maria Casagrande

**Affiliations:** 1 Department of Dynamic and Clinical Psychology and Health Studies, “Sapienza” University of Rome, 00185 Rome, Italy; renata.tambelli@uniroma1.it (R.T.); maria.casagrande@uniroma1.it (M.C.); 2 Department of Psychology, Temple University, Philadelphia, PA 19122, USA; eunice.chen@temple.edu

**Keywords:** eating behaviors, overweight, executive functions, food stimuli

## Abstract

Human nutrition is characterized by both automatic and self-regulated processes. One of the dimensions that may be employed in monitoring eating behaviors is the cognitive control played by the executive functions (EFs). The role of EFs in affecting eating behaviors has been assessed in empirical studies, but further analyses are needed in a current society characterized by a food-abundant environment. Accordingly, this study attempted to evaluate the association between weight status and executive functions in response to food-related stimuli. One hundred and forty-four young adults took part in the study (25.7% in overweight condition). The participants completed a set of computerized cognitive tasks to assess cognitive and motor inhibition and working memory in two different conditions: (i) classic versions of the tasks and (ii) modified versions with food cues. The results indicate that food stimuli may influence executive performance and that there is an association between food cue-related executive functioning, particularly in the domain of motor inhibition, and working memory. These results suggest that self-regulation in nutrition may involve executive control. Although further studies are needed, this work suggests the importance of a multidimensional perspective in the analysis of eating behaviors and the relevance of EFs in monitoring our approach to food stimuli in an environmental context.

## 1. Introduction

Eating and nutrition in humans were considered for a long time exclusively automatic behaviors, primary for human survival. However, they are now defined as composed of both automatic and self-regulated components. The current perspective highlights the important roles of the environment, educational programs, and cognitive dimensions in controlling and monitoring such behaviors [1]. Considering the environmental and social role, studies have reported that, from the acquisition of food autonomy, food availability, social influences, and cultural context impact food intake [2]. Furthermore, personological characteristics, including traits, affects, emotional regulation, and stress management, are also found to affect eating habits, both in terms of decreased and increased food intake [3,4]. From a cognitive perspective, in human behaviors, executive functions (EFs)—cognitive processes that control and drive thoughts and goal-directed behaviors (e.g., cognitive inhibition, working memory, and shifting; see refs. [5,6])—play an important role as moderators in the relationship between intention and execution. Accordingly, they should be considered in the context of the self-regulated side of eating behavior.

The role of EFs in affecting eating behavior has been empirically investigated in clinical populations affected by eating disorders (EDs), in which neurocognitive deficits involving EFs were confirmed [7,8,9]. If this aspect is true in EDs, it is even more important to detect it in healthy eating behaviors because preference toward markers of healthy food consumption patterns (i.e., low in fats, sugar, and calories) and markers of unhealthy food consumption patterns (i.e., hypercaloric food, characterized by high sugar and fats) affects food intake and consequently influences weight gain and physical and health status [10,11,12]. Consequently, the relationship between environmental factors (e.g., food stimuli) and individual characteristics (e.g., executive functioning and response to food stimuli) represents a crucial area for further investigation in the field of nutrition.

In the current society, characterized by a food-abundant environment, the response to food stimuli may contribute to the tendency to overeat [13]. In fact, some authors have reported that individuals who showed an overeating behavior exhibited an increased approach towards food stimuli compared to non-food stimuli, perceived as salient and arousing, and this approach is associated with inhibitory deficits or loss of control [12,14,15]. An altered response toward food stimuli has been reported in conditions of excessive body weight [10,15]. For example, Stingl and colleagues [16] demonstrated that individuals with obesity exhibited worse working memory performance, as assessed by a modified version of the n-back task adopting food stimuli, compared to individuals with a healthy weight condition. Kulendran et al. [17] reported an improvement in cognitive inhibition, as evaluated with a modified version of the Stroop task with food stimuli, in a sample of individuals with severe obesity following bariatric surgery. Price and colleagues observed poor response inhibition, assessed by a food-based Go/No-Go task in response to food stimuli in a sample of adults with overweight/obesity with low dietary restraint compared to individuals with normal weight [18]. Moreover, in a sample of healthy undergraduate students, Calitri and colleagues found a predictive role of the performance in a Food Stroop task, using both healthy and unhealthy food stimuli, on body weight increase [19]. However, other studies on obesity [20,21,22] did not present the same results, probably due to heterogeneity in methods [10,23], which does not clarify the actual role of food stimuli in the relationship between executive functions and eating behavior or weight condition. Indeed, the discordance in previous studies on the association between executive functions and body weight may have been driven by the different versions of the tasks in measuring the same domain [10].

Considering previous research, this study aimed to clarify the role of food-related stimuli in the relationship between executive functions and weight status, adopting modified versions of EF tasks [10]. To this end, a sample of healthy young adults with no eating disorder or significant overweight (i.e., BMI ≤ 30) was considered. The difference in performance between healthy weight and moderate overweight was assessed using executive tasks with food stimuli.

The specific aims of the study were as follows:

(a) To test the association between classic and modified versions of executive tasks and to justify the adoption of tasks using food stimuli in the field of nutrition and eating habits.
(b) To evaluate the association between the performance in the executive tasks adopting food stimuli, especially if high-palatable, and body weight condition assessed by the body mass index (BMI) [13,24], expecting that executive performance can predict an increase in BMI.
(c) To verify the differences between normal weight and overweight conditions in executive performance toward food stimuli. We expect a greater difficulty in individuals who are overweight than those with normal weight condition in controlling the inhibitory responses toward food-related stimuli.

## 2. Materials and Methods

### 2.1. Participants

The participants were recruited from the pool of students of “Sapienza” the University of Rome. The dissemination of the project in the laboratory social networks and a snowball procedure allowed to recruit participants on a voluntary basis in the last months of 2019. Specific exclusion criteria were the presence of psychiatric conditions, EDs, or chronic illness (e.g., diabetes, metabolic syndrome, and neurological pathologies). Moreover, individuals undergoing pharmacological treatments that may affect cognitive performance in executive tasks were excluded.

### 2.2. Outcomes

#### 2.2.1. Demographic Information

A semi-structured face-to-face interview collected the participants’ main demographic information (gender, age, and level of education) and medical and clinical history in order to assess inclusion and exclusion criteria.

#### 2.2.2. Physiological Measures

The body mass index (BMI) was computed by dividing weight by height (meters squared) assessed in a laboratory setting by the researchers. Weight conditions were classified according to WHO guidelines (normal weight: BMI < 25 Kg/m^2^; overweight BMI > 25 Kg/m^2^) [25]. In addition to BMI, two further descriptive indices of body condition were included: the waist-to-height ratio (W/h) [26] and body adiposity index (BAI = ((hip circumference)/((height)1.5) − 18)) [27]. The assessment was carried out according to the procedure suggested by the Center for Diseases Control in its guideline [28].

#### 2.2.3. Executive Functions

Table 1 shows similarities and differences among the two versions of the task adopted in the study.

### 2.3. Classic Tasks for Executive Functions

**Figure 1:**
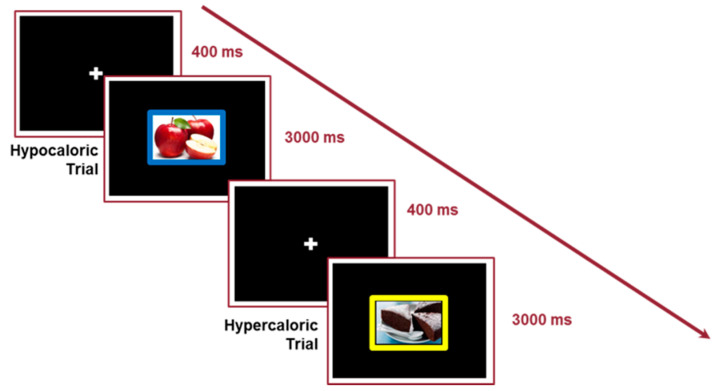
Picture emotional Stroop food cue procedure.

A computerized version of the Stroop task [29] was adopted to assess cognitive inhibition and interference control [30]. The task provided the administration of target stimuli consisting of colored words (font: Courier New; font size: 60; colors: yellow, red, blue, and green) that were semantically related to the colors YELLOW, RED, BLUE, and GREEN. Each word could be presented with the ink color related to its semantic meaning (Congruent Condition) or another color (Incongruent Condition). The task required the subject to press the key corresponding to the ink color as quickly and accurately as possible. Following a practice block, a block of 120 trials (60 Congruent and 60 Incongruent, presented in random order) was presented. An initial fixation cross (duration: 400 ms) was presented before each trial. The target stimulus remained on the screen for 3000 ms or until the participant’s response. Reaction times (RTs) of correct responses were collected, and the Stroop effect (mean RT Incongruent Trials—mean RT Congruent Trials) was computed. The procedure and stimuli are shown in Figure 1.

The Go/No-Go Task [31] allowed to assess motor inhibition, i.e., the ability to control an inadequate motor response. The stimuli consisted of two geometric shapes, each measuring 960 × 720 pixels, placed in the center of the screen with a black background. The Go stimulus was a green circle, and the No-Go stimulus was a green triangle. A fixation cross (duration: 500 ms) was presented, followed by the presentation of target stimuli (Go) and non-target stimuli (No-go), in a randomized order considering three, four, or five Go trials for each No-Go trial. After a practice block, the procedure provided 2 blocks of 50 trials each (n = 100). The inappropriate responses to “no-go” stimuli were summed to define the number of False Alarms.

The n-back task [32] was used to assess working memory. The stimuli consisted of some alphabetical letters presented in the center of a white screen (font size: 30; font: Palatino Linotype). A sequence of one-by-one stimuli (duration: 2500 ms) was presented, followed by a blank screen with an interstimulus interval (ISI) of 500 ms. Two sessions of the task were presented in sequence: the one-back and the two-back procedure. In the one-back session (n trials = 40), the participant was required to evaluate whether each stimulus was the same as the previous one (Target) or different (Non-Target). In the two-back session (n trials = 40), the participant had to indicate whether the stimulus was the same or different from the stimulus presented in the two previous trials. In each block, 30% of the trials were Target. The percentage of accuracy for both one-back and two-back procedures was considered a measure of working memory.

### 2.4. Modified Version of the Tasks with Food Stimuli

The Picture Emotional Stroop Food Cue [33] was adopted to evaluate the interference control as an inhibition index. The task provided the administration of target stimuli consisting of colored pictures (300 × 300 pixels) from three different categories: objects (Neutral Condition; n = 6), high-palatable food (Hypercaloric Condition; n = 6), and low-palatable food (Hypocaloric Condition; n = 6). The neutral stimuli were drawn from the International Affective Picture System [34]. The food stimuli were pictures of traditional Western meals selected from a database of 61 images. These images were assessed by an independent sample of 54 undergraduate students, who rated them on a 0 to 10 rating scale for valence, palatability, and satiety. A high score for any of the three indices indicated hypercaloric food (score higher than 7.5), while a low score indicated hypocaloric food (score lower than 5.0). Each image had a colored frame. The colors were those used in the Stroop task (i.e., YELLOW, RED, BLUE, and GREEN). Six additional images were constituted by a colored frame with no image in the center (Color Condition). Each stimulus was randomly presented on the screen in one of the four possible color frames (in balanced order). The task required responding as quickly and accurately as possible by pressing the key corresponding to the color of the frame. The experiment was introduced by a practice block of 16 trials with feedback about correct execution. Afterward, two blocks of 144 randomly presented trials (24 for each category) were presented. An initial fixation cross (duration: 400 ms) was presented before each trial. The target stimulus remained on the screen for 3000 ms or until the participant’s response. According to the aim of the study, mean reaction times (RTs) and the percentage of accuracy for each condition were calculated, considering the correct responses with RTs included between 200 and 2000 ms [13]. Moreover, the Conflict Effect Indices for the Hypercaloric and Hypocaloric conditions (mean RTs in Hypercaloric Trials—mean RTs in Neutral and Color Trials; mean RTs in Hypocaloric Trials—mean RTs in Neutral and Color Trials) were calculated for each participant. Figure 1 shows an example of the task procedure.

**Figure 2:**
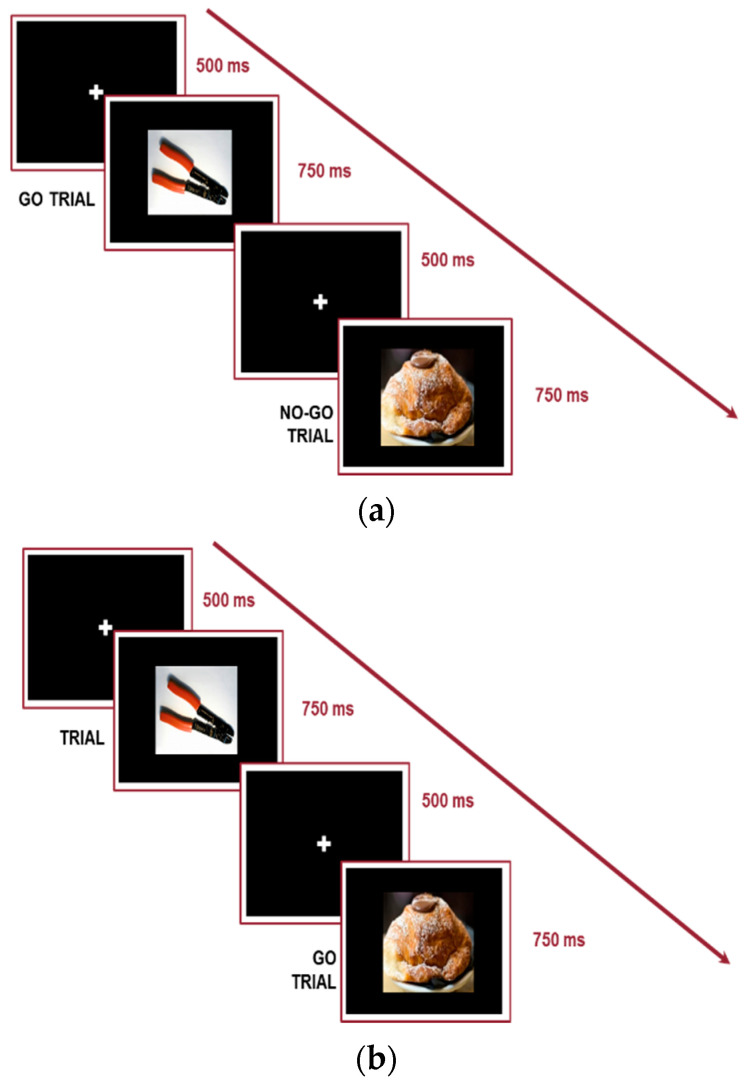
Food Cue Go/No-Go Task. (**a**) Food No-Go version; (**b**) Do-it-yourself tools of the No-Go version.

The Food Cue Go/No-Go Task [18] was developed to assess motor inhibition in response to food stimuli. The stimuli consisted of two categories of pictures: neutral (do-it-yourself tools) selected from the International Affective Picture System [34] and high-palatable food selected from the same database utilized for the Stroop task. Each picture was presented on a black background in the center of the screen. Two versions of the procedure were administered in a counterbalanced order between the participants. In one version of the procedure, the food stimuli represented the Go condition (No-Go = do-it-yourself tools), while, in the other version, the food stimuli represented the No-Go condition. The participant was required to maintain fixation on the center of the screen throughout the experiment. The initial screen with a fixation cross (duration: 500 ms) was followed by the presentation of target stimuli (Go) and non-target stimuli (No-go), which were presented in a randomized order considering three, four, or five Go trials for each No-Go trial. Each stimulus remained in the center of the screen for 750 ms or until the participant’s response. The task required to press the left mouse key as quickly as possible when the picture associated with the Go condition appeared in the center of the screen. When the No-Go picture appeared, the participant had to wait for the disappearance of the stimulus. Each version of the task comprised 200 trials divided into two blocks of 100 trials each. A practice block of 12 trials, with feedback on correctness, was presented at the beginning of the experiment. The inappropriate responses to “no-go” trials (36 for each Go/No-Go task) were summed to define the number of False Alarms. The percentages of False Alarms ((no-go incorrect responses/total number of no-go trials) × 100) on the Go Food and No-Go Food versions of the task were adopted as an index of the motor component of inhibition. An accuracy greater than 50% was accepted for including the participant in the analysis. The procedure and stimuli are shown in Figure 2.

**Figure 3:**
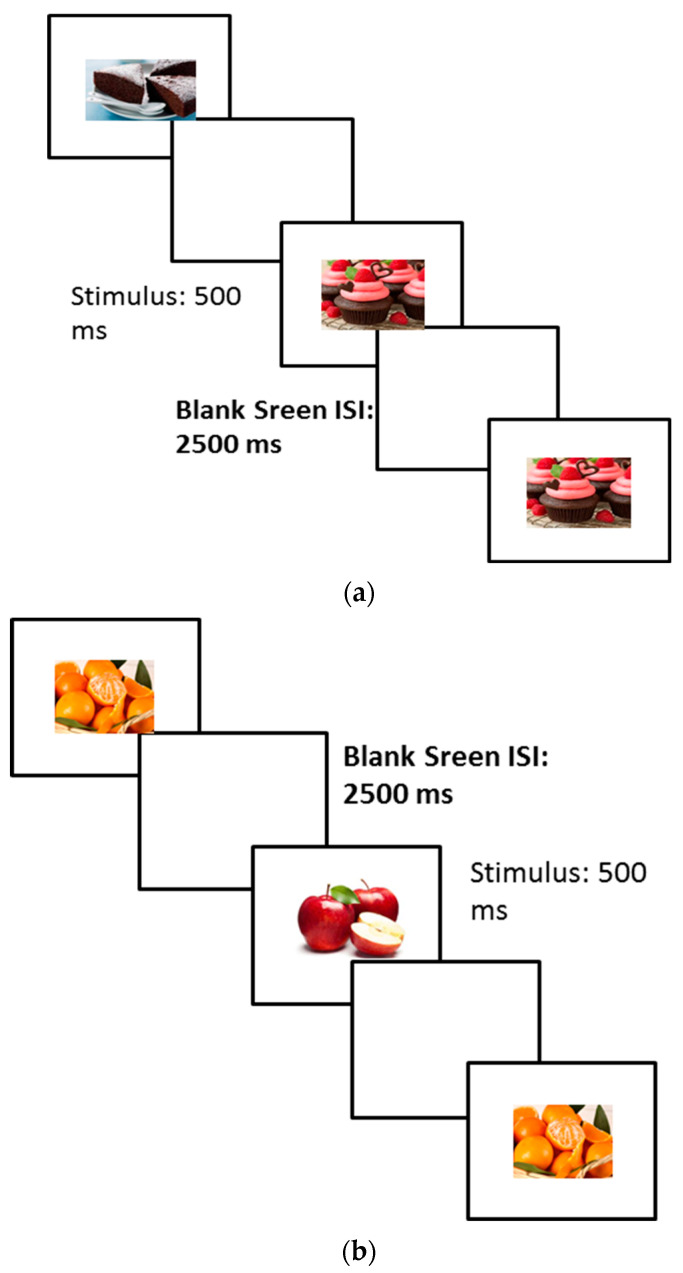
Example of procedure of 1-back and 2-back tasks. (**a**) Hypercaloric 1-back trials; (**b**) hypocaloric 2-back trials.

The Visual 1-Back and 2-Back Food Cue [16] were developed to assess working memory performance related to food cues. The stimuli comprised images of hypercaloric and hypocaloric food (extracted from the same validated database used for the other tasks), presented on a white background in the center of the screen. The experiment consisted of two balance conditions according to the valence of the stimuli (Hypercaloric and Hypocaloric). For each task condition, two sessions were presented in sequence: the one-back and the two-back. A sequence of one-by-one stimuli (duration: 500 ms) was presented, followed by a blank screen (ISI: 2500 ms). In the one-back session, the participant was required to evaluate whether each stimulus was the same (Target) or different (Non-Target) from the previous stimulus. In the two-back session, the participant had to indicate whether the stimulus was the same or different from the stimulus presented in the two previous trials. The responses were collected by pressing the key “X” for the target stimuli and the key “M” for the non-target stimuli. The Hypercaloric and Hypocaloric procedures include 40 trials for the one-back block and 40 trials for the two-back blocks. In each block, 30% of the trials were Target. The percentage of accuracy for both the one-back and two-back procedures was considered an index of working memory performance. The procedure and stimuli are shown in Figure 3.

### 2.5. General Procedure

The research was conducted according to the Helsinki Declaration, and the Local Ethics Committee (Department of Dynamic and Clinical Psychology and Health Studies—”Sapienza” the University of Rome; protocol n. 0000450, 15 April 2019) approved it. The written informed consent was explained, and the participants signed it before the evaluation.

Each participant was individually tested in a silent, dimly illuminated room with a comfortable temperature, and the procedure was explained before the in-person interview. For each computerized version of the cognitive tasks, a practice block was included to familiarize the participant with the task. Finally, the participants’ weight, height, and blood pressure were measured, and the experiments were administered.

### 2.6. Data Analysis

Means and standard deviations (i.e., continuous variables) and frequency and percentage (i.e., categorical variables) were collected for descriptive variables and performance on the tasks.

One-way ANOVAs were conducted to compare the two groups (Normal weight; Overweight) for each descriptive variable and cognitive performance included in the study.

To verify the association between classical tasks and food cue tasks, linear Pearson’s r correlations were calculated between the Stroop task (Stroop Effect on RTs), the Go/No-Go Task (% of False Alarms), 1- and 2-Back tasks (% of Accuracy), and the indices of the Picture Emotional Stroop Food Cue (RTs in Hypercaloric and Hypocaloric stimuli and the Hypercaloric and Hypocaloric Effect), the Food-Cue Go/No-Go (% of false alarms for food no go trials; % of false alarms for do-it-yourself trials), and the 1-Back and 2-Back Food Cue (percentage of accuracy).

To verify the predictive role of the performance in the executive tasks adopting food cues for the BMI, single linear regression models were calculated for each task, using as control linear regression models considering as predictors the indices of the classical tasks.

Finally, mixed ANOVAs were employed to verify for each task the role of the food cues and the type of food cue in influencing the differences in the executive performance in the two groups of participants.

For all the statistical analyses, the level of significance was set at *p* < 0.05. Statistical analyses were conducted using Jamovi^®^ (ver. 2.3.21.0).

## 3. Results

### 3.1. General Information

The overall sample of the participants comprised one hundred and forty-four university students (age range: 18–30 years; mean age = 23.48 ± 2.85; 55 males, 89 females). A total of 25.7% of the sample (37 out of 144 participants) presented an overweight condition in the absence of a clinical diagnosis of EDs or obesity in anamnestic assessment (BMI range: 25–30; mean BMI = 27.19 ± 2.86). The main characteristics of the overall sample are shown in Table 2. To test for differences in performance in the executive tasks between normal-weight and overweight conditions, a convenience sample included all participants with overweight (N: 37; 18 males; 19 females; mean BMI = 27.19 ± 2.86) and a sub-group of participants with normal weight (N: 40; 20 males, 20 females; mean BMI = 21.63 ± 1.88), balanced for age and gender. The main characteristics of the participants are shown in Table 3.

### 3.2. The Relationship between Classical Task and Task Adopting Visual Food Stimuli

The linear Pearson’s r correlations between classical and food cue tasks in the overall sample are shown in Table 4. The indices of the different versions of the Go/No-Go task were found to be positively correlated (Classical Go Food: r = 0.56; *p* = 0.0001; Classical No-Go Food: r = 0.51; *p* = 0.0001), as well as the indices of the 2-Back tasks (Classical Hypercaloric: r = 0.57; *p* = 0.0001; Classical Hypocaloric: r = 0.47; *p* = 0.0001). Moreover, the 1-Back classical version was positively correlated with the 1-Back with hypocaloric cues (r = 0.37; *p* = 0.0001). It is noteworthy that the Stroop task did not show a significant Pearson’s r correlation with the Picture Emotional Stroop Food Cue. This result suggests that the two tasks, despite often being reported as tasks assessing inhibitory control, may actually measure different executive components (see Table 4).

### 3.3. The Association between BMI and Executive Functions Associated with Food Stimuli

To verify the single association between EF performance and BMI changes, different regression models were calculated. Specifically, faster RTs toward food cues (both Hypercaloric and Hypocaloric) in the Stroop task and higher accuracy in both the two versions of the 2-Back task (Hypercaloric and Hypocaloric) are associated with a higher BMI (see Table 5). The Stroop effect (R^2^ = 0.002; t = 1.14; *p* = 0.26), the percentage of false alarms (R^2^ = 0.002; t = 0.46; *p* = 0.26), and the 1-Back (R^2^ = 0.01; t = −1.17; *p* = 0.26) and 2-Back accuracies (R^2^ = 0.001; t = −0.16; *p* = 0.87) were not found to be associated with the BMI.

### 3.4. Analyses of Variance (Normal-Weight Group vs. Overweight Group)

**Figure 4:**
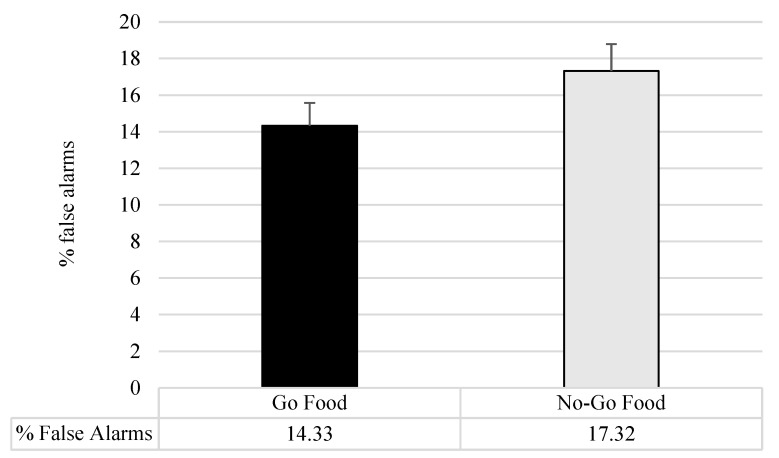
Mean and std. err of the performance in the two versions of Food Cue Go/No-Go tasks. Go Food: % of false alarms for do-it-yourself tools (no-go procedure); No-Go Food: % of false alarms for food cue (No-Go procedure).

Considering the classical version of the executive tasks, no significant differences emerged between the two groups (see Table 6). Similar results were found by the mixed ANOVAs on the executive task adopting food cues, with no significant between-group differences (F < 1; *p* > 0.20). However, when considering the Picture Emotional Stroop Food Cue, a significant effect of the stimuli was highlighted (F_3,230_ = 9.96; *p* = 0.0001; pη2 = 0.12) with faster reaction times in the color frame condition compared to the picture conditions (vs neutral: t = 3.09; *p* = 0.003; vs hypercaloric: t = −4.00; *p<* 0.0001; vs hypocaloric: t = −4.86; *p* = 0.0001), while no differences emerged between the three picture valences. No within-subject difference was present in the elaboration of the food cue stimuli compared to the neutral one. The mixed ANOVA on the two versions of the Food Cue Go/No-Go reported a significant effect of the task (F_1,75_= 9.70; *p* = 0.003; pη2 = 0.14). Generally, the participants reported a higher percentage of false alarms in the motor inhibition toward the food stimuli (Food-No-Go) than toward the neutral stimuli (Food-Go) (see Figure 4). The Group and the Group x Task interaction were not significant (F < 1; *p* > 0.60). The analyses on the two versions of the visual 1-Back and 2-Back did not show significant effects for the group, the type of food, or the group x type of food interaction (F < 1; *p* > 0.22).

## 4. Discussion

Within the theoretical framework in which human eating behavior is self-regulated with a role of the brain frontal areas and executive control [30], this study attempted to evaluate the association between weight status and executive functions in an environment characterized by food stimuli. Many studies reported the importance of sensitivity toward food and food cue as factors influencing eating behaviors and weight gain [35], identifying the role of rewarding of palatable stimuli and the effect on the behavioral response of these aspects.

To achieve this main objective, this study has assessed the reliability of different versions of tasks adopted in the literature to assess EFs. To the best of our knowledge, this is the first study to investigate the association between environmental stimuli (i.e., food cues) and cognitive functions (i.e., executive functioning), attempting to control the performances considering the functioning independently of food cues and performance in response to food cues. Moreover, according to the most prevalent theoretical models on EFs, our study assessed the three core dimensions of executive functioning (cognitive inhibition, motor inhibition, and working memory) [6]. The results indicate a positive correlation between the classical and food cue versions of the Go/No-Go task and the 2-Back task, suggesting that the use of different stimuli and task procedures allow for the evaluation of the same executive domains. According to Miyake’s model, these results indicate the possibility of evaluating a single EF independently by the type of stimuli used [5]. However, a different result was found for the Stroop tasks. The Stroop task has been considered the gold standard for measuring the inhibitory process [36]. The modified version of the Stroop, defined as Emotional Stroop for the impact of emotional activating stimuli (pictures or words), has previously been adopted to assess the inhibitory process in an emotional context [36,37,38]. However, the studies yielded inconsistent results regarding the suitability of the Stroop task for this purpose due to the possible interference of cognitive processes on another [39]. This controversy may explain the dissociation between our two versions of the Stroop task. As suggested by Nijs et al. [13] and other authors [19], modified versions of the Stroop task may be more suitable for defining attentional bias toward specific stimuli rather than an executive cognitive inhibition. Consequently, the results of this study confirm this assumption.

The second objective of this study was to confirm the association between the executive response toward food and weight condition as an indirect expression of eating habits and food preference [13,30]. Despite the low reliability of the regression models, our results indicate that the reaction times in the Stroop task using food-related stimuli (both hypocaloric and hypercaloric) and the accuracy in the 2-Back task (both with hypercaloric and hypocaloric stimuli) would predict the BMI. Specifically, our findings on the n-back task are inconsistent with those of previous studies that have reported a decline [16] or no difference [21] in working memory performance when food stimuli were used to compare obesity and normal-weight conditions. Conversely, our results agree with those of Rutters and colleagues, who have observed that food stimuli are strongly represented in the working memory system, thereby facilitating the performance on the task [40]. These findings are consistent with the assumption indicating that the high reactivity to food could influence working memory [41]. Accordingly, we might hypothesize that a greater attraction for food stimuli may be associated with a higher BMI. The attraction for food stimuli can influence overweight through the working memory, which may modulate visual attention toward food and the consequent eating behavior [40]. This last aspect allows interpreting the results related to the Picture Emotional Stroop Food Cue. If the previous results indicated that the Stroop with food stimuli would evaluate attentional mechanisms related to cognitive control rather than cognitive inhibition, and if working memory could play a role in the processing of food-related stimuli, as suggested by Rutters [40], then the “fast reaction times—high BMI” association in the case of food stimuli could be determined by the interaction between working memory and cognitive control. It should be noted that this pattern occurs in both highly palatable and hypocaloric stimuli, confirming the salient role of food regardless of its palatability [23,42]. However, this interpretation should be taken with caution, and further studies should test the mediating and moderating effect of the relationship between these variables in larger and better-distributed samples.

The final aim of the study was to analyze the differences between normal-weight and overweight conditions in executive functioning in response to food cues. A systematic review of the literature [10] highlighted a general bidirectional relationship between excessive body weight and EFs, indicating that an executive impairment is associated with a more severe overweight condition. However, this review identified several limitations in the existing literature. Firstly, there is a paucity of studies investigating the early stage of overweight (BMI < 30). Secondly, the heterogeneity in methodologies employed does not lead to clear results. Moreover, the literature suggested that the role of food cues in executive functions’ performance and the differences between diverse weight conditions remain unclear [43,44]. Contrary to our hypothesis, no differences were observed between individuals with overweight and normal weight in both the classical and modified versions of the tasks. Moreover, the Picture Emotional Stroop Food Cue revealed no between-group or within-subject differences in food-cue elaboration. Nevertheless, an interesting preliminary result was found considering the two versions of the Go/No-Go task. A within-subject difference was observed between the Go Food and No-Go food versions of the task. Specifically, a higher motor inhibition was reported in response to palatable food stimuli (in a task with many food stimuli) than neutral stimuli (in a task with few food stimuli). This result agrees with previous studies that have reported a similar inhibitory control pattern assessed by a Go/No-Go task in individuals with obesity and normal-weight conditions, suggesting that the salience of stimuli influences individuals with different weight conditions in a similar way [22]. Moreover, the results indicate that the inhibitory response in an environment with a high density of food stimuli may result in a greater difficulty in inhibiting overeating behavior. However, future studies are needed to clarify this aspect.

Although this study yielded interesting preliminary results and is the first to integrate and compare different versions of cognitive tasks to assess the executive response toward food stimuli and its association with weight status, some limitations should be highlighted. As previously suggested, future studies should consider mediating and moderating models to verify the relationships highlighted by these preliminary results, particularly with regard to the association between cognitive control, working memory, and the BMI. The limited sample size and the relatively low percentage of overweight individuals (25.7 percent of the sample) prevented us from implementing this analysis design. Furthermore, the low percentage of overweight individuals certainly reduced the statistical power of the results. The limitation of the sample should also be emphasized with respect to the comparison of the two groups via convenience sampling. Despite this study reporting a similar and sometimes higher sample size than previous studies on this topic [13,16,19,22], it is essential to acknowledge this potential impact on the reliability of the findings. However, given that this study focused on a healthy population with mild overweight levels, some results may not have emerged due to the relatively small sample size. Another aspect that can be considered both a limitation and a starting point for future studies concerns the poor theoretical background on this topic, making this study preliminary both in its methodological framework and in the results obtained. Once again, the importance of standardizing experimental protocols to verify the role of food-related stimuli on executive functioning should be emphasized, as evidenced by studies on attentional bias [10]. Moreover, given the inconsistent results observed in the studies on the relationship between executive functions and overweight in the absence of pathological conditions, further studies should also focus more on this relationship across the human lifespan [10]. Finally, we must consider the importance to replicate these results in order to verify how the relationship between weight and cognitive functions may be altered across populations in recent years characterized by the pandemic experience that has been reported as a factor affecting weight condition and increasing overweight and obesity prevalence, as well as an experience affecting at some degree cognitive functions [45,46,47].

## 5. Conclusions

In conclusion, the results of this study indicate that the executive response to food stimuli may play a role in weight gain. These findings contribute to a better understanding of the processes underlying BMI increases associated with dysfunctional eating behaviors. Moreover, shifting the perspective from a view of eating as an automatic process to one in which individuals’ self-regulation plays a role in determining food intake may have practical implications for preventing more severe overweight conditions [48]. Considering executive functions as a set of cognitive functions that monitor behaviors, but that are also influenced by our emotional state, affective condition, and psychological characteristics, represents another significant milestone in this kind of study. This suggests the importance of a multidimensional evaluation of eating behaviors and, eventually, eating dysregulated behavior conditions. Counteracting the weight gain observed during the initial stages of overweight could help to reduce the negative trend of obesity prevalence that has been observed in recent years [49], stratified in all different cultures and all social and age groups, and has been identified by the WHO as a significant issue for the future generations.

## Figures and Tables

**Table 1:** Main characteristics of the cognitive tasks.

		Classic Version	Modified Version
Stroop Task	Stimuli Types	Words of color	Colored Pictures
	Target Response	Color of the words	Color of the frame of the picture
	Conditions	Congruent; Incongruent	Hypercaloric Food; Hypocaloric Food; Neutral No Food.
	Fixation cross time	400 ms	400 ms
	Target stimulus duration	3000 ms	3000 ms
	Number of Trials	120 (60 incongruent; 60 congruent)	288 (96 for each condition)
Go/No-Go Task	Stimuli Types	Geometric shapes (green circle; green triangle)	Colored Pictures
	Target Response	No-Go = no response; Go = response	No-Go = no response; Go = response
	Conditions	No-Go = green triangle	Version 1: No-Go = Food stimuli Version 2: No-Go = do-it-yourself stimuli
	Fixation cross time	500 ms	500 ms
	Target stimulus duration	750 ms	750 ms
	Number of Trials	100	Version 1: 100 Version 2: 100
N-Back Task	Stimuli Types	Letters	Colored Pictures
	Target Response	1-Back: Target stimulus is previous one 2-Back: Target stimulus is two previous one	1-Back: Target stimulus is previous one 2-Back: Target stimulus is two previous one
	Conditions	Target (30% of the stimuli): Letter condition	Target (30% of the stimuli): Hypercaloric Trials; Hypocaloric Trials.
	Blank Screen	2500 ms	2500 ms
	Target stimulus duration	500 ms	500 ms
	Number of Trials	1-Back: 40 2-Back: 40	1-Back: 40 2-Back: 40

**Table 2:** Characteristics of the overall sample.

	N (%)
Demographic Information	
Sex	
Males	55 (38)
Females	89 (62)
Lifestyles Habits	
Smoking Habits	
Yes	55 (38)
No	89 (62)
Caffeine Consumption	
Yes	109 (76)
No	35 (24)
Alcohol Consumption	
Yes	81 (56)
No	63 (44)
Physical Activity	
Yes	70 (49)
No	74 (51)
Health Risk Factors: Family diseases (yes)	
Dementia/Mild Cognitive Impairment	28 (19)
Diabetes	68 (47)
Obesity	27 (19)
Cardiovascular Disorders	70 (49)
Hypertension	56 (39)

**Table 3:** Characteristics of the sample classified according to body weight condition and ANOVA results.

	Normal Weight	Overweight	F	*p*	Pη2
N (m/f)	40 (20/20)	37 (18/19)			
Age (mean, sd)	24.33 (1.76)	24.73 (2.68)	<1	0.43	0.01
Years of education (mean, sd)	17.10 (1.66)	16.62 (1.85)	1.43	0.24	0.02
Physiological Measures (mean, sd)					
Weight (kg)	65.12 (9.36)	81.53 (14.79)	34.10	0.0001	0.31
Height (m)	1.73 (0.10)	1.73 (0.11)	<1	0.97	0.00001
BMI	21.93 (1.88)	27.19 (2.86)	103.54	0.0001	0.58
Waist-to-height ratio	0.45 (0.04)	0.51 (0.05)	35.75	0.0001	0.34
Body adiposity index	26.20 (4.71)	32.09 (5.71)	22.66	0.0001	0.25
Systolic blood pressure	119.58 (10.79)	119.86 (10.77)	<1	0.91	0.00001
Diastolic blood pressure	71.68 (8.33)	74.25 (7.28)	2.04	0.16	0.03
Heart rate	78.05 (14.01)	75.46 (11.03)	<1	0.38	0.01

**Table 4:** Pearson’s r correlation between classical executive tasks and food cue executive tasks.

		1	2	3	4	5	6	7	8	9	10
Stroop Effect	r	0.04	0.02	0.05	0.02	−0.0002	0.04	0.07	0.12	−0.17	−0.17
False Alarms (%) Go/No-Go	r	0.14	0.08	−0.17	−0.21	0.56 ***	0.51 ***	−0.24	−0.20	−0.11	−0.12
1-Back Task	r	0.09	0.13	0.001	0.10	−0.17	−0.09	0.11	0.36 ***	0.001	0.27 ***
2-Back Task	r	−0.25 **	−0.28 ***	−0.09	−0.21 *	−0.01	−0.01	0.04	0.10	0.57 ***	0.47 ***

1 = RT_Hypercaloric; 2 = RT_Hypocaloric; 3 = Hypercaloric_Effect; 4 = Hypocaloric_Effect; 5 = Food Go (% of false alarms in do-it-yourself tools trials); 6 = Food No-Go (% of false alarms in food cue trials); 7 = 1-Back Hypercaloric; 8 = 1-Back Hypocaloric; 9 = 2-Back Hypercaloric; 10 = 2-Back Hypocaloric. * *p* < 0.05; ** *p* < 0.01; *** *p* < 0.001.

**Table 5:** Regression models with EF performance as predictors and BMI as a dependent variable.

Models	R^2^_adj_	F	B	SE	beta	*p*	Zero-Order Correlation
RT_Hypercaloric stimuli	0.03	3.77	−0.01	0.004	−0.18	0.05 *	−0.18
RT_Hypocaloric stimuli	0.03	3.97	−0.01	0.003	−0.18	0.05 *	−0.18
Food Go	−0.01	<1	−0.006	0.03	−0.02	0.96	−0.02
Food No-Go	−0.003	<1	0.02	0.03	0.08	0.40	0.08
1-Back hypercaloric	0.02	3.83	4.87	2.49	0.18	0.05 *	0.18
1-Back hypocaloric	0.01	2.68	5.17	3.15	0.15	0.10	0.15
2-Back hypercaloric	0.03	4.08	3.67	1.82	0.18	0.05 *	0.18
2-Back hypocaloric	0.03	4.13	3.60	1.77	0.19	0.04 *	0.19

SE = standard error. * *p* ≤ 0.05.

**Table 6:** Performance in the executive task of the two groups of participants.

	Normal Weight	Overweight	F	*p*	Pη2
Classical Executive Tasks					
Stroop Task Reaction Times					
Congruent condition	689.34 (73.94)	682.74 (78.60)	<1	0.71	0.001
Incongruent condition	759.11 (87.01)	760.29 (93.60)	<1	0.96	0.00001
Stroop effect	69.78 (50.25)	77.55 (49.03)	<1	0.50	0.01
Stroop Task % of accuracy					
Congruent condition	96.40 (0.92)	96.94 (0.97)	<1	0.68	0.002
Incongruent condition	95.25 (6.81)	94.86 (5.67)	<1	0.79	0.001
Go/No-Go Task					
% False Alarms	9.62 (7.64)	12.14 (7.62)	2.02	0.16	0.03
1-Back and 2-Back					
1-Back % Target Accuracy	94.97 (8.87)	93.86 (11.47)	<1	0.37	0.01
2-Back % Target Accuracy	81.80 (17.61)	85.44 (14.05)	<1	0.33	0.01
Executive Tasks with Food Cue					
Picture Emotional Stroop Food Cue Reaction Times					
Neutral Cue	712.23 (89.61)	695.48 (84.89)	<1	0.43	0.01
Color Cue	688.36 (93.24)	690.83 (87.79)	<1	0.91	0.00001
Hypercaloric Cue	709.18 (87.77)	700.59 (76.61)	<1	0.67	0.003
Hypocaloric Cue	721.17 (102.19)	693.22 (84.20)	1.52	0.22	0.02
Hypercaloric Effect	−8.93 (34.29)	−7.44 (48.71)	<1	0.88	0.0001
Hypocaloric Effect	−20.93 (7.43)	6.54 (7.42)	4.45	0.04	0.06
Picture Emotional Stroop Food Cue % of accuracy					
Neutral Cue	96.43 (0.45)	97.03 (0.50)	<1	0.37	0.01
Color Cue	96.73 (0.43)	98.74 (0.49)	2.39	0.13	0.03
Hypercaloric Cue	97.75 (0.60)	96.29 (0.68)	<1	0.62	0.004
Hypocaloric Cue	97.25 (0.61)	95.45 (0.70)	1.89	0.17	0.03
Hypercaloric Effect	−0.18 (3.40)	1.11 (3.6)	2.34	0.13	0.03
Hypocaloric Effect	−0.15 (3.00)	1.93 (3.81)	6.49	0.01	0.09
Food Cue Go/No-Go Task % False Alarms					
Food Go Task	13.65 (8.73)	14.73 (10.92)	<1	0.66	0.003
No-Go Task	16.20 (11.28)	16.94 (11.74)	<1	0.79	0.001
Visual 1-Back and 2-Back Food Cue % of Target accuracy					
1-Back Hypercaloric	94.68 (15.95)	96.19 (6.63)	<1	0.62	0.004
1-Back Hypocaloric	95.32 (6.70)	95.62 (6.70)	<1	0.85	0.001
2-Back Hypercaloric	76.10 (21.72)	82.09 (16.24)	1.68	0.19	0.02
2-Back Hypocaloric	78.20 (20.19)	82.31 (15.21)	<1	0.34	0.01

## Data Availability

The original contributions presented in the study are included in the article, further inquiries can be directed to the corresponding author.
